# Developmental Trajectories of Anxiety and Depression Symptoms from Early to Middle Childhood: a Population-Based Cohort Study in the Netherlands

**DOI:** 10.1007/s10802-019-00550-5

**Published:** 2019-05-08

**Authors:** Jasmijn M. de Lijster, Michiel A. van den Dries, Jan van der Ende, Elisabeth M.W.J. Utens, Vincent W. Jaddoe, Gwendolyn C. Dieleman, Manon H.J. Hillegers, Henning Tiemeier, Jeroen S. Legerstee

**Affiliations:** 1 Department of Child and Adolescent Psychiatry/Psychology, Erasmus MC – Sophia Children’s Hospital, Erasmus University Medical Center, P.O. Box 2060, Wytemaweg 80, 3000 CB Rotterdam, the Netherlands; 2 The Generation R Study Group, Erasmus University Medical Center, Rotterdam, The Netherlands; 3 Research Institute of Child Development and Education, University of Amsterdam, Amsterdam, The Netherlands; 4 Academic Center for Child Psychiatry the Bascule /Department of Child and Adolescent Psychiatry, Academic Medical Center, Amsterdam, The Netherlands; 5 Department of Epidemiology, Erasmus University Medical Center, Rotterdam, The Netherlands; 6 Department of Pediatrics, Erasmus University Medical Center, Rotterdam, The Netherlands; 7 Department of Social and Behavioral Sciences, Harvard T.H. Chan School of Public Health, Boston, MA USA

**Keywords:** Anxiety and depression symptoms, Developmental trajectories, Early childhood, Growth mixture modeling, Psychosocial outcomes, School outcomes

## Abstract

Developmental patterns of anxiety and depression symptoms in early childhood have previously been related to anxiety and mood disorders in middle childhood. In the current study, trajectories of anxiety and depression symptoms (1.5–10 years) were related to children’s broader psychosocial and school-related functioning at 10 years. We included a population-based sample of 7499 children, for whom primary caregivers reported anxiety and depression symptoms on the Child Behavior Checklist, at children’s ages of 1.5, 3, 6, and 10. Growth Mixture Modeling identified four distinct, gender-invariant, trajectories of anxiety and depression symptoms: low (82.4%), increasing (7.4%), decreasing (6.0%), and increasing symptoms up to age 6 followed by a decrease to age 10 (preschool-limited, 4.2%). Children with a non-Dutch ethnicity had lower odds to be in the increasing trajectory and higher odds to be in the decreasing and pre-school limited trajectory. Also, low maternal education predicted the decreasing and pre-school limited trajectory. Higher levels of psychopathology during pregnancy for both mothers and fathers predicted the increasing, decreasing, and preschool-limited trajectory, compared to the low trajectory. At age 10, children in the increasing and preschool-limited trajectory had diminished psychosocial outcomes (friendship-quality and self-esteem) and worse school-related outcomes (school performance and school problems). This study adds to current knowledge by demonstrating that developmental patterns of anxiety and depression symptoms in early childhood are related to broader negative outcomes in middle childhood. Child and family factors could guide monitoring of anxiety and depression symptoms in the general population and provide targets for prevention programs.

During early childhood, anxiety and depression symptoms are common. Studies have shown that up to 14% of preschool-aged children have clinical levels of anxiety and depression (Bayer et al. 2008; Egger and Angold 2006). Anxiety and depression during childhood are linked to a broad range of negative outcomes, including emotional disorders at a later age (Goodwin et al. 2004; Roza et al. 2003). In addition, symptoms of anxiety and depression have been related to social problems and diminished school functioning. More specifically, anxiety and depression symptoms have been negatively associated with children’s daily functioning; such as lower performance at elementary school (Ialongo et al. 1994, 1995; Kovacs and Goldston 1991; Muris and Meesters 2002) and a diminished social competence, peer acceptance, and friendship quality (Kingery et al. 2010; Rudolph et al. 1994).

Children with high levels of anxiety and depression symptoms in early childhood are more likely to experience these symptoms in middle childhood (Bayer et al. 2010; Mesman et al. 2001; Mian et al. 2011). However, the developmental course of anxiety and depression symptoms in children from the general population is likely to follow different patterns (Broeren et al. 2013; Cote et al. 2002). Mapping differences in developmental trajectories of anxiety and depression symptoms might help to identify children who are at risk of developing psychosocial problems before clinical anxiety and depression manifest. Group-based trajectory modeling is a valuable statistical approach to identify different courses of anxiety and depression in early childhood (Curran et al. 2010). This approach is able to identify sub-groups with shared common patterns of change in anxiety and depression over time (Fanti and Henrich 2010; Jung and Wickrama 2008).

Several previous population-based studies have indeed identified trajectories of anxiety and depression symptoms across early childhood and related these to adverse outcomes. Feng et al. (2008) identified four trajectories between the ages of 2 and 10 of anxiety symptoms in a small sample of boys from low-income families. Decreasing as well as increasing trajectories of anxiety were associated with an anxiety or mood disorder at the age of 10 years. Sterba et al. (2007) and Fanti and Henrich (2010) identified three trajectories from early to middle childhood: a low, a moderate, and a high anxiety and depression trajectory. Most of the children were assigned to the low trajectory. Both studies showed that the trajectories of high anxiety and depression symptoms were associated with problem behavior, such as self-reported depression by the age of 11 years (Sterba et al. 2007) and parent-reported peer problems at the age of 12 years (Fanti and Henrich 2010). However, in the general population, the relation between developmental trajectories of anxiety and depression symptoms with broader psychosocial and school functioning in childhood has, to our knowledge, not been investigated. Studying associations between anxiety and depression trajectories and psychosocial and school functioning is valuable because these domains are important indicators of children’s quality of life and psychological well-being (Almquist 2012; Masselink et al. 2018).

More insight into predictors of trajectories could further help to understand which children are at risk of developing anxiety and depression symptoms from early to middle childhood. Several child and family characteristics have been related to trajectories of anxiety and depression symptoms in early childhood. For instance, gender has been identified as a risk factor for the longitudinal course of anxiety and depression symptoms (Cote et al. 2002, 2009; Sterba et al. 2007). In addition, developmental trajectories of anxiety and depression symptoms have been consistently associated with maternal psychopathology in previous studies (Cote et al. 2009; Feng et al. 2008; Sterba et al. 2007). Although the role of paternal psychopathology in the development of anxiety and depression symptoms has been acknowledged (Bogels and Phares 2008), this effect has not been taken into account when predicting trajectories. Economic disadvantage has been associated with children’s internalizing symptoms in early childhood (Bradley and Corwyn 2002; Rijlaarsdam et al. 2013). However, inconsistent results have been found for socioeconomic disadvantage as a risk factor for increasing levels of anxiety and depression symptoms (Cote et al. 2009; Fanti and Henrich 2010). As socioeconomic disadvantage is associated with parental psychopathology (Lancaster et al. 2010), both parental characteristics should be taken into account to estimate their relative contribution in predicting children’s trajectories of anxiety and depression symptoms.

The present study examined predictors of developmental trajectories of anxiety and depression from early to middle childhood as well as the effect of these trajectories on psychosocial (friendship quality and self-esteem) and school-related outcomes (school performance and school problems). Previous studies have examined parent-ratings of children’s psychosocial functioning as outcomes of trajectories. Although parent-ratings can approximate children’s experiences, these measures are not well suited to replace self-reports (Jonsson et al. 2017). We used data from the Generation R Study; a large, population-based cohort including repeated measures across childhood and detailed information regarding outcomes in middle childhood from both the child and parents perspective. Aims of this study were: (1) to examine developmental trajectories of anxiety and depression symptoms from early to middle childhood, (2) to identify predictors associated with these trajectories, and (3) to examine associations between anxiety and depression symptom trajectories and psychosocial and school-related outcomes. Based on previous studies, we hypothesized to identify up to four different trajectories of anxiety and depression symptoms with most children having constant low symptoms. We further hypothesized low socioeconomic status and parental psychopathology to be associated with increasing trajectories of anxiety and depression. In addition, we hypothesized worse psychosocial and school-related outcomes for children whose symptoms increase from early to middle childhood.

## Method

### Participants

This study was embedded in the Generation R Study, an ongoing multi-ethnic population-based prospective cohort from fetal life onward in Rotterdam, the Netherlands. The Generation R Study is designed to identify early causes of normal and abnormal growth, development, and health. Its design has been previously described in detail (Kooijman et al. 2016). In short, all pregnant women living in Rotterdam, with an expected delivery date between April 2002 and January 2006, were invited to participate and contacted via obstetrician practices. The study was approved by the Medical Ethics Committee of the Erasmus Medical Center. Anonymity was guaranteed and written informed consent was obtained from children’s primary caregivers at each study phase.

In total, 9778 mothers enrolled in the study gave birth to 9749 live-born children. During the preschool period (0–4 years), parents of the 1166 children living outside the definite study area at birth were not approached as the logistics of postnatal follow-up were embedded in the municipal routine child care system. When all parents were contacted again when children were 5 years, consent for their children to participate was 85% of the original sample (Jaddoe et al. 2012). Data on children’s anxiety and depression symptoms were available at ages 1.5 years (*n* = 5223), 3 years (*n* = 4939), 5 to 7 years (*n* = 6210; hereafter referred to as ‘age 6’) and 10 years (*n* = 4938). In total, the study sample comprised of 7499 children with anxiety and depression data available for one or more research assessment(s) (76.2% response rate). For psychosocial and school-related outcomes, the study sample varied across outcomes (*n* = 4336 friendship quality, *n* = 4355 self-esteem, *n* = 3669 school performance, and *n* = 3857 school problems).

### Measures

#### Child Anxiety and Depression Symptoms

Child anxiety and depression symptoms were assessed with the Child Behavior Checklist (CBCL). The CBCL /1½-5 was sent to all primary caregivers to be completed at home around the time of the research assessments at the ages 1.5, 3, and 6 years. For the assessment at 5 to 7 years, both versions of the CBCL could have been used. As it was anticipated that the majority of children were younger than 6 years at the time of assessment, the CBCL/1½-5 was used for all children to assure a uniform assessment at this age. The CBCL/6–18 was used at the assessment of 10 years (Achenbach and Rescorla 2001). Good reliability and validity have been reported for both versions of the CBCL across different populations (Achenbach and Rescorla 2000, 2001). At all ages, the CBCL was completed by primary caregivers, which were generally mothers. For this study, we used the empirically derived Anxious/Depressed subscale. The Anxious/Depressed scale comprises eight items in the CBCL/1½-5 and 13 items in the CBCL/6–18. Correlations of the Anxious/Depressed subscale over time followed an autoregressive pattern with values between *r* = 0.16, *p* < 0.001 (1.5 years and 10 years) and *r* = 0.44, *p* < 0.001 (1.5 and 3 years).

Internal consistency (reliability) was measured with categorical omega’s (ω_c_) because of the categorical-ordered items in all questionnaires in this study (Kelley and Pornprasertmanit 2016). In addition, we report 95% CIs to illustrate the uncertainty of these estimates. For the CBCL Anxious/Depressed scale, internal consistency in the current study was 0.62, 95% CI [0.61, 0.64] at age 1.5 years, 0.70, 95% CI [0.68, 0.71] at age 3 years, 0.75, 95% CI [0.73, 0.76] at age 6 years and 0.82, 95% CI [0.81, 0.82] at age 10 years, which is comparable to normative samples (Achenbach and Rescorla 2000, 2001).

#### Predictors of Anxiety and Depression Symptom Trajectories

Socioeconomic status was measured by children’s ethnicity and mother’s highest completed educational level at study enrollment. Children were classified as non-Dutch if one of the parents was born abroad. Ethnicity was defined into three categories: Dutch, other Western, and non-Western. Mothers educational level was classified into three categories: *low* (primary school or lower vocational education), *intermediate* (intermediate vocational education), and *high* (higher vocational education or university). Further, at study enrollment, parental psychopathology symptoms were assessed with the use of the Brief Symptom Inventory (BSI; Derogatis 1993; De Beurs 2004). The BSI is a widely used screening tool for general psychopathology with excellent reliability and validity (Boulet and Boss 1991; De Beurs and Zitman 2006). Levels of parental psychopathology in the Generation R sample are in correspondence with data from the manual by de Beurs (2009) and other population-based studies found the same percentages of mothers (6–7%) and fathers (2–3%) scoring above the cut off of general psychopathology during pregnancy (Kjeldgaard et al. 2017; Kvalevaag et al. 2013). To determine the level of psychopathology, we used the Global Severity Index (GSI) based on the total 53 items of the BSI. Scores on the GSI were standardized into z-scores to facilitate the interpretation of these predictor variables. Internal consistency (ω_c_) was 0.97, 95% CI [0.97, 0.97] for fathers and 0.99, 95% CI [0.99, 0.99] for mothers.

#### Psychosocial Functioning (Age 10)

##### Friendship Quality

We used an adapted version of the Friendship Quality Questionnaire (FQQ; Parker and Asher 1993). The FQQ has been validated using socio-metric rating methods and is predictive of both peer acceptance and feelings of loneliness (Parker and Asher 1993). This first questionnaire for children in Generation R comprised multiple domains and was completed at home. Therefore, 10 items of the original 40-item FQQ were selected based on expert opinion and relevance to the Dutch elementary school setting. Items represented subscales ‘validation and caring’ (i.e. *we give each other compliments*), ‘companionship’ (i.e. *we are always together during our break at school*), ‘conflict resolution’ (i.e. *if we are angry at each other, we always talk it out*), ‘intimate exchange’ (i.e. *we tell each other secrets*), and ‘help and guidance’ (*if we need to get something done, we will help each other*). Children rated how true each statement was about their best friend (1 = *not true*, 2 = *somewhat true*, 3 = *very true,* total range 10–30). Missing values were replaced by the mean score of the remaining items (weighted total score). If there were more than 20% of the answers missing, this was coded as a missing value. Internal consistency (ω_c_) of the adapted FQQ in this study was 0.70, 95% CI [0.68, 0.71].

##### Self-Esteem

Children’s self-esteem was measured with an adapted question format of the Harter’s Self Perception Profile for Children (SPPC) corresponding to Wichstrom (1995) containing 18 items of the original SPPC (CBSK in Dutch; Veerman et al. 1997). Answers were given on an adapted scale (1 = *not true*, 2 = *somewhat true*, 3 = *very true,* total range 18–54). A weighted total score was created for statistical analysis when at least 14 items were completed. The CBSK has acceptable reliability and validity (Dongen-Melman et al. 1993) and has been shown to be gender-invariant (Van den Bergh and Van Ranst 1998). In this study, internal consistency (ω_c_) of the modified version of the CBSK was 0.81, 95% CI [0.80, 0.82].

#### School Functioning (Age 10)

##### School Performance

We used the school performance scores from the CBCL/6–18 at age 10 years. The child’s performance was rated across four major academic subjects: ‘reading or language’, ‘history’, ‘arithmetic or math’, and ‘geography’ (1 = *failing*, 2 = *below average*, 3 = *average*, 4 = *above average*, and *not applicable,* total range 4–20). A weighted total score was created for statistical analysis when at least three items were completed. When a subject was not applicable to a child, we treated that score as a missing value. Internal consistency (ω_c_) of the child’s school performance score was 0.74, 95% CI [0.73, 0.74].

##### School Problems

School problems were measured through three items from the CBCL/6–18 mother report at age 10, which describe possible difficulties that a child might experience during school. These were the following three questions: 1 ‘*Does your child receive special or remedial services or attend a special class or special school?*’, 2 ‘*Has your child repeated any grade?*’, and 3 ‘*Has your child had any academic or other problems in school?*’. The total school problems score was dichotomized to no school problems versus ≥1 school problems.

### Statistical Methods

Categorical omega’s for internal consistency were computed with the MBESS R package version 4.4.3 (Kelley 2018). Descriptive analyses were conducted with SPSS version 21.0 (IBM Corp. 2016). All other analyses were performed with Mplus version 8 (Muthén and Muthén 2017). We used growth mixture modeling (GMM) to determine trajectories of anxiety and depression symptoms in early childhood (1.5–10 years). To be able to model these trajectories, average scores on the Anxious/Depressed scale of the CBCL/1 ½-5 and CBCL/6–8 from the four assessments were used when at least 75% of the items were completed. GMM is a statistical technique that can be used to find subgroups of change in individuals over time while allowing for within-class or trajectory variability (Fanti and Henrich 2010; Jung and Wickrama 2008).

To identify the best model fit, we compared models with different trajectory solutions with the use of both the Bayesian information criterion (BIC) and the Lo-Mendell-Rubin test (LMRT). A lower BIC score indicates a model with a better fit and the LMRT tests indicates whether a given model has a significantly better fit than a solution with one trajectory less. In addition, a plot of trajectories for each model was produced to see whether these solutions were distinctive in capturing the development of anxiety and depression symptoms. Each model was fitted with a linear slope and with a quadratic slope in addition. Classification accuracy was determined by both mean assignment probabilities for each trajectory and overall model entropy. Entropy with values approaching one indicate a clear delineation of trajectories (Celeux and Soromenho 1996). Because of the low internal consistency of the CBCL Anxious/Depressed subscale at age 1.5 years, we compared trajectories with four assessments from 1,5 to 10 years with trajectories based on the sample of children from the three assessments 3, 6, and 10 years (*n* = 7130).

We then examined gender invariance by determining whether the best fitting model in the total group was valid for both boys and girls. Invariance was examined by testing whether trajectories had the same growth parameters (mean, variance and covariance of the intercept, linear slope, and quadratic slope) across gender (Sterba et al. 2007). This was done by simultaneously estimating the models for both boys and girls and using Wald tests to examine differences in growth parameters between boys and girls.

After determining the final model, we examined the association between possible predictors of anxiety and depression symptom trajectories by entering the predictors as auxiliary variables in the growth mixture model. We simultaneously fitted the trajectories and the associations of predictors in a multinomial logistic regression model. As maternal age has been related to increased levels of psychological stress during pregnancy (Aasheim et al. 2012), maternal age was included as a predictor of anxiety and depression symptom trajectories as well.

Then, we examined the associations of anxiety and depression symptom trajectories with psychosocial and school-related outcomes using the three-step approach with an auxiliary model (Asparouhov and Muthén 2014). This approach uses classification probabilities together with the most likely trajectory to correct for classification errors when examining the association of the trajectories with outcomes. For continuous outcomes, differences in the mean and for dichotomous outcomes, the thresholds were compared across trajectories while adjusting for the predictors of these anxiety and depression symptom trajectories. A threshold model was used to test differences for dichotomous outcomes; thresholds are expected values that have to be exceeded for a person to be in a particular category (Muthén and Muthén 2017). Because of the high comorbidity between internalizing and externalizing problems (Thomas and Guskin 2001), all outcomes analyses were repeated while adjusting for externalizing problems at age 10 years. We used global Wald model tests for differences in outcomes across trajectories. When significant, subsequent follow-up Wald model tests were performed to determine which trajectories contributed to the overall difference in outcomes. This hierarchical approach reduces the number of independent tests.

#### Missing Data

GMM is able to account for missing values within the growth trajectories using Full Information Maximum Likelihood (FIML) with all data that is available. Thus, missing values on anxiety and depression symptoms were taken into account by FIML for the model decision and gender invariance analyses. In addition, missing data on possible predictors of anxiety and depression symptom trajectories were imputed with the use of multiple imputation. Percentages of missing data were 1.7% for child ethnicity, 8.0% for maternal education, 25.8% for maternal and 41.4% for paternal psychopathology symptoms. For this, 20 imputed datasets were generated by using a fully conditional specified model.

## Results

### Sample Characteristics

Table 1 gives an overview of the descriptive characteristics of the children and their parents in the study sample and the original Generation R sample. Children from the study sample most often had a Dutch or non-Western ethnicity and the majority of the mothers had an intermediate or higher educational level. Most mothers and fathers reported low levels of parental psychopathology. Mean levels of anxiety and depression symptoms for children in the study sample were 0.15 (*SD* = 0.18) at 1.5 years, 0.13 (*SD* = 0.19) at 3 years, 0.18 (*SD* = 0.23) at 6 years, and 0.17 (*SD* = 0.20) at 10 years.

**Table 1:** Sample characteristics of the study sample (*n* = 7499) and original Generation R sample (*N* = 9749)

Characteristics	Study sample *n* = 7499	Original sample *N* = 9749
Child gender, %		
Boys	49.5	49.3
Girls	50.5	50.7
Child ethnicity, %		
Dutch	58.7^a^	53.8
Other Western	9.0	8.9
Non-Western	32.3	37.3
Maternal education, %		
High	48.8^a^	42.9
Intermediate	30.1	30.6
Low	21.0	26.4
Maternal age, mean (SD)	30.7 (5.04)^a^	29.9 (5.37)
Maternal psychopathology symptoms, mean (SD)	0.27 (0.35)^a^	0.30 (0.38)
Paternal psychopathology symptoms, mean (SD)	0.14 (0.22)^a^	0.15 (0.24)

^a^Characteristic significantly different in study sample

### Non-response Analyses

The sample characteristics of the study sample (*n* = 7499) were compared with the characteristics of children and their parents from the original sample who did not participate (*n* = 2250). Children in the study sample were more likely to be Dutch (χ^2^ = 426.96, *p <* 0.001) than those not participating. Mothers of children from the study sample were more likely to have a high level of education (χ^2^ = 689.50, *p <* 0.001) and be older (*t* = 25.20, *p* < 0.001). Psychopathology during pregnancy of participating mothers (*t* = −11.02, *p* < 0.001) and fathers (*t* = −5.99, *p* < 0.001) in the study, was lower compared to parents who were not participating.

### Model Selection and Model Fit

Fit indices for models with 2 to 5 number of latent classes are presented in Table 2. The 4 class trajectory model solution with a quadratic slope had the best fit. Although the BIC for the model with 5 classes showed a slightly better fit, the 5 class solution did not lead to another distinct trajectory. Additionally, the LMRT value indicated that the more parsimonious model with 4 trajectory classes provided a better fit than the 5 class model. For the model with 4 latent classes, a model with a quadratic slope showed a better fit (BIC = −14,196.50) compared to a 4 class model with only a linear slope (BIC = −13,797.95). Classification accuracy of the 4 class model was good with an entropy value of 0.85 and average latent class probabilities ranging from 0.74 to 0.95. The same 4 class solution came forward for the sample that included measurements at 3, 6, and 10 years (*n* = 7130), supporting the inclusion of the measurement of anxiety and depression symptoms at 1.5 years despite lower internal consistency. As a 4 class model with a random linear slope and/or random quadratic slope did not converge for the gender-invariance analyses, a model with a random intercept only was chosen as a final model for subsequent analyses.

**Table 2:** Comparison of model fit for different latent class trajectories of anxiety and depressive symptoms (*n* = 7499)

Model	#	%	Trajectories	BIC	LMRT *p* value	Entropy	Average latent class probability
2	1	0.910	Low	−12,049.08	0.001	0.90	0.88
	2	0.090	Increasing to decreasing				0.98
3	1	0.851	Low	−13,439.29	< 0.001	0.86	0.95
	2	0.077	Increasing				0.87
	3	0.072	Decreasing				0.85
4	1	0.824	Low	−14,196.50	0.064	0.85	0.82
	2	0.074	Increasing				0.95
	3	0.060	Decreasing				0.74
	4	0.042	Pre-school limited				0.86
5	1	0.746	Low	−14,718.07	0.125	0.81	0.75
	2	0.131	Increasing to medium				0.90
	3	0.057	Decreasing				0.87
	4	0.038	Pre-school limited				0.73
	5	0.028	Increasing to high				0.84

### Developmental Trajectories of Anxiety and Depression Symptoms

**Fig. 1:**
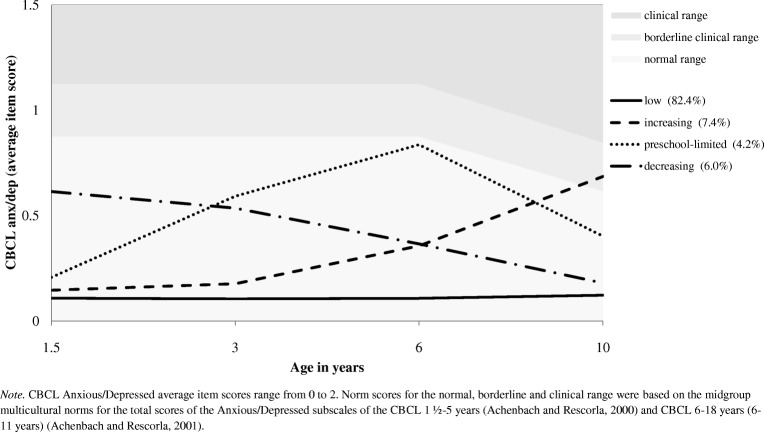
Developmental trajectories of anxiety and depression symptoms (*n* = 7499)

Figure 1 gives a graphical presentation of the four anxiety and depression symptom trajectories. We defined the steady low anxiety and depression slope over time (solid line) as the *low trajectory*. Most children were within this trajectory (82.4%). The decreasing anxiety and depression slope over time (dashed-dotted line) comprised 6.0% of the children (*decreasing trajectory)*. The slope that shows increasing anxiety and depression symptoms up to 6 years and a decreasing slope from 6 to 10 years (dotted line), was defined as the *preschool-limited trajectory* and comprised 4.2% of the children. The increasing anxiety and depression slope over time (dashed line) comprised 7.4% of the children (*increasing trajectory*). Anxiety and depression symptoms for children in this trajectory increased to borderline clinical levels at age 10. To examine whether differences between the trajectories were driven by certain items of the CBCL Anxious/Depressed scale, we conducted in-depth analyses of discrepancies in anxiety and depression items between the trajectories. Eight overlapping items that were assessed in both versions of the CBCL showed the same pattern of trajectories as presented in Fig. 1. Thus, differences between trajectories were driven by age and the number of symptoms, instead of specific items.

### Gender Invariance

With regard to differences in trajectories across gender, invariance held for the 4 class model solution. The 4 class model elicited the same type of trajectories for both boys (*n* = 3785) and girls (*n* = 3714). The means of the intercepts, linear slopes, quadratic slopes and variance of the intercept of the 4 trajectories were equal across gender (all *p* > 0.16). We, therefore, concluded that the anxiety and depression symptom trajectories were gender invariant. Small gender differences in the distribution of proportions across categories were observed within the trajectories. The percentage of boys was higher in the low trajectory (82.6%) and pre-school limited trajectory (3.9%) than the percentage of girls (77.3% and 2.9%, respectively). In contrast, the percentage of boys was lower in the increasing (7.3% boys vs. 8.4% girls) and decreasing (6.1% boys vs. 11.4% girls) trajectory compared to girls.

### Predictors of Anxiety and Depression Symptom Trajectories

An overview of the associations between predictors and anxiety and depression symptom trajectories is presented in Table 3. Most strongly, children with a Western ethnicity or non-Western ethnicity had higher odds to be in the decreasing trajectory relative to the low trajectory, OR = 4.41, 95% CI [2.21, 8.82], OR *=* 8.79, 95% CI [5.10, 15.16], than Dutch children. Also, children with a non-Western ethnicity had higher odds to be in the pre-school limited trajectory, OR = 1.64, 95% CI [1.14, 2.34]. In contrast, children with a Western or non-Western ethnicity had lower odds to be in the increasing trajectory relative to the low trajectory, OR = 0.57, 95% CI [0.33, 1.00], OR = 0.68, 95% CI [0.48, 0.97]. Children of mothers with low educational level had higher odds to be in the decreasing, OR *=* 2.02, 95% CI [1.28, 3.16] as well as in the preschool-limited trajectory, OR = 1.57, 95% CI [1.02, 2.41], compared to children of mothers with high educational level. Maternal psychopathology was positively associated with the increasing trajectory, OR = 1.77, 95% CI [1.50, 2.10], the decreasing trajectory, OR *=* 1.62, 95% CI [1.36, 1.93], and the preschool-limited trajectory, OR *=* 1.80, 95% CI [1.55, 2.09]. A similar pattern was found for paternal psychopathology being positively associated with the increasing trajectory, OR = 1.24, 95% CI [1.05, 1.46], decreasing, OR = 1.24, 95% CI [1.07, 1.45], and preschool-limited trajectory, OR = 1.29, 95% CI [1.12, 1.49].

**Table 3:** Adjusted associations between predictors and anxiety and depression symptoms trajectories (*n* = 7499): the increasing, decreasing, preschool-limited trajectory compared with the low trajectory

Anxiety and depressive symptoms trajectories										
Predictors	Low (ref)	Increasing			Decreasing			Preschool-limited		
	OR	OR	95% CI	*p* value	OR	95% CI	*p* value	OR	95% CI	*p* value
Child gender										
Boys	ref	1.00			1.00			1.00		
Girls		1.18	0.90–1.54	0.352	0.91	0.67–1.22	0.511	1.03	0.77–1.39	0.841
Child ethnicity										
Dutch	ref	1.00			1.00			1.00		
Western		**0.57**	**0.33–1.00**	**0.049**	**4.41**	**2.21–8.82**	**< 0.001**	1.00	0.55–1.83	0.990
Non-Western		**0.68**	**0.48–0.97**	**0.032**	**8.79**	**5.10–15.16**	**< 0.001**	**1.64**	**1.14–2.34**	**0.007**
Maternal education										
High	ref	1.00			1.00			1.00		
Intermediate		0.75	0.52–1.07	0.109	1.07	0.66–1.75	0.774	0.92	0.60–1.40	0.690
Low		0.84	0.56–1.25	0.398	**2.02**	**1.28–3.16**	**0.002**	**1.57**	**1.02–2.41**	**0.042**
Age mother		0.99	0.96–1.02	0.653	0.99	0.96–1.01	0.323	1.00	0.97–1.03	0.925
Maternal psychopathology		**1.77**	**1.50–2.10**	**< 0.001**	**1.62**	**1.36–1.93**	**< 0.001**	**1.80**	**1.55–2.09**	**< 0.001**
Paternal psychopathology		**1.24**	**1.05–1.46**	**0.011**	**1.24**	**1.07–1.45**	**0.005**	**1.29**	**1.12–1.49**	**< 0.001**

Maternal and paternal psychopathology represent z-scores. Significant predictors (*p* < 0.05) are printed in bold

### Psychosocial Functioning

The associations of anxiety and depression symptom trajectories with friendship quality and self-esteem are presented in Table 4. *Friendship quality* varied between the four trajectories (*Wald* = 15.63, *p* = 0.001). Children in the increasing and preschool-limited trajectory had lower friendship quality compared to children in the low trajectory (*d* = −0.15). After adjusting for externalizing problems at 10 years, self-reported friendship quality did no longer differ across the four trajectories (*Wald* = 6.52, *p* = 0.089).

**Table 4:** Differences in adjusted means of psychosocial and school-related outcomes per trajectory at the age of 10 years

	Psychosocial outcomes								School-related outcomes							
	Friendship quality^b,f^				Self-esteem^c^				School performance^d^				School problems^e^			
Trajectory	Mean^a^	*Wald*	*d*	*p* value	Mean^a^	*Wald*	*d*	*p* value	Mean^a^	*Wald*	*d*	*p* value	Thres-hold^a^	*Wald*	*d*	*p* value
Low (ref)	23.43	–	–	–	46.72	–	–	–	14.31	–	–	–	0.74	–	–	–
Increasing	22.57	**7.71**	**- 0.15**	**0.006**	39.05	**173.33**	- **0.83**	**< 0.001**	13.46	3.55	- 0.15	0.060	- 0.55	**44.39**	- **0.36**	**< 0.001**
Decreasing	22.75	2.40	- 0.12	0.121	46.99	0.67	0.05	0.413	14.84	**9.00**	**0.17**	**0.003**	0.42	1.51	- 0.09	0.220
Preschool-limited	22.67	**4.23**	**- 0.15**	**0.040**	46.05	1.87	- 0.10	0.171	14.17	0.24	- 0.04	0.626	- 0.06	**12.50**	**- 0.24**	**< 0.001**
Low	23.43	2.40	0.12	0.121	46.72	0.67	- 0.05	0.413	14.31	**9.00**	- **0.17**	**0.003**	0.74	1.51	0.09	0.220
Increasing	22.57	0.12	- 0.03	0.733	39.05	**156.22**	- **0.94**	**< 0.001**	13.46	**8.39**	- **0.26**	**0.004**	- 0.55	**9.37**	- **0.30**	**0.002** ^**f**^
Decreasing (ref)	22.75	–	–	–	46.99	–	–	–	14.84	–	–	–	0.42	–	–	–
Preschool-limited	22.67	0.89	- 0.02	0.895	46.05	2.44	- 0.18	0.119	14.17	**3.87**	- **0.25**	**0.049** ^**f**^	- 0.06	1.96	- 0.16	0.161
Low	23.43	**4.23**	**0.15**	**0.040**	46.72	1.87	0.10	0.171	14.31	0.24	0.04	0.626	0.74	**12.50**	**0.24**	**< 0.001**
Increasing	22.57	0.05	- 0.02	0.830	39.05	**83.80**	**- 0.80**	**< 0.001**	13.46	1.40	- 0.13	0.237	- 0.55	2.77	- 0.17	0.096
Decreasing	22.75	0.89	0.02	0.895	46.99	2.44	0.18	0.119	14.84	**3.87**	**0.25**	**0.049** ^**f**^	0.42	1.96	0.16	0.161
Preschool-limited (ref)	22.67	–	–	–	46.05	–	–	–	14.17	–	–	–	- 0.06	–	–	–

Significant differences (*p* < 0.05) between mean values are printed in bold

^a^Means and thresholds were adjusted for child’s gender, ethnicity, maternal age, education and maternal and paternal psychopathology

^b^*n* = 4336

^c^*n* = 4355

^d^*n* = 3669

^e^*n* = 3857

^f^No longer significant after adjusting for children’s externalizing problems at 10 years of age

Further, different levels of *self-esteem* were observed between the trajectories (*Wald* = 181.51, *p* < 0.001). Children in the increasing trajectory had a lower self-esteem than children in the other three trajectories (*d* = −0.80 to −0.94). For self-esteem, the same results were found after adjusting for externalizing problems (*Wald* = 96.54, *p <* 0.001).

### School Functioning

Table 4 also presents the results of the associations of anxiety and depression symptom trajectories with school performance and school problems. *School performance* of the children varied across trajectories (*Wald* = 14.05, *p* = 0.003). Children with a decreasing trajectory of anxiety and depression symptoms had a higher school performance (*d* = 0.17) compared to children in the low trajectory. Children within the increasing trajectory and the preschool-limited trajectory had worse school performance compared to children in the decreasing trajectory (*d* = −0.25, *d =* −0.26). Although the model test for school performance remained significant after adjusting for externalizing problems (*Wald* = 12.56, *p =* 0.006), school performance between children in the pre-school limited trajectory and decreasing trajectory no longer differed (*Wald* = 2.98, *p* = 0.084).

*School problems* of the children varied across trajectories (*Wald* = 57.15, *p* < 0.001). Children within the increasing trajectory had a lower threshold for school problems compared to children in the low and decreasing trajectory (*d* = −0.30, *d =* −0.36). Children within the preschool-limited trajectory had a lower threshold for school problems compared to children in the low class (*d = −*0.24). School problems varied across trajectories after adjusting for externalizing problems (*Wald* = 15.56, *p <* 0.001). However, the same thresholds for school problems were found for children in the increasing and decreasing trajectory (*Wald* = 3.76, *p* = 0.052).

## Discussion

This study examined developmental trajectories of anxiety and depression symptoms from early to middle childhood in a large population-based cohort. For children between the ages of 1.5 and 10 years old, four distinct trajectories of anxiety and depression symptoms were found. These increasing, preschool-limited, decreasing, and low trajectories were predicted by child and family characteristics that were identifiable before anxiety and depression symptoms developed. Both the increasing and preschool-limited trajectories were associated with lower self-esteem and poorer school-related outcomes at 10 years old when externalizing symptoms were taken into account. This study, therefore, shows that developmental patterns of anxiety and depression symptoms in early childhood are related to negative psychosocial and school outcomes in middle childhood.

The four trajectories identified in the current study are partly in line with the results of previous studies from early to middle childhood. As expected within a population-based cohort, most children (82.4%) experienced low levels of anxiety and depression symptoms. Previous studies also found similar patterns of increasing and decreasing symptoms in this age group (Fanti and Henrich 2010; Feng et al. 2008). However, our study also shows a unique trajectory, which is not established before. This trajectory was apparent for a small proportion (4.2%) of children who experienced an increase in anxiety and depression symptoms up to the age of 6, followed by a decrease of these symptoms by the age of 10 years. Both moderate and steep trajectories of increasing symptoms have previously been reported for children between the ages of 1.5 and 5 years (Cote et al. 2009). The results of our study indicate that increasing symptoms show two patterns after the age of 5, namely a preschool-limited trajectory and a persistent increasing trajectory. An explanation for the preschool-limited trajectory could be that these symptoms represent fears that are specific and developmentally related (Broeren et al. 2013). Also, this trajectory could be explained by comorbid externalizing symptoms after the age of 6 that may become more visible and result in underreporting of internalizing symptoms (Thomas and Guskin 2001). Studies on internalizing and externalizing problems have shown that a subsample of children shows persistent co-morbidity between these symptoms between the ages of 2 and 12 (Fanti and Henrich 2010). However, as children in the pre-school limited trajectory remained to have more school problems than children in the low trajectory after adjusting for externalizing problems, other factors than co-morbidity may explain this trajectory.

Identifying which children are more likely to follow a certain trajectory of anxiety and depression symptoms may help predict their further course of symptoms in middle childhood. Previous studies defined gender as a risk factor of increasing anxiety and depression symptom trajectories (Cote et al. 2009) or specified trajectories for boys and girls separately (Cote et al. 2002; Sterba et al. 2007). In contrast to previous studies, gender did not explain differential trajectories when adjusting for other child and family characteristics. In addition, our gender invariance analyses showed that the trajectories for boys and girls were comparable. An explanation for the discrepancy between our gender-invariance results and those found by Sterba et al. (2007), could be the small difference in the children’s maximum age of the samples. Children in the study by Sterba and colleagues were followed up from 2 years until the age of 11 and gender differences for the increasing trajectories in their study became greater with increasing age. In the current study, these differences were not found between boys and girls up to the age of 10. Most probably, differences in the developmental course of anxiety and depression might become more apparent during puberty (Legerstee et al. 2013).

Both child and family characteristics in this study predicted differential trajectories of anxiety and depression symptoms. Consistent with previous research, maternal psychopathology was a risk factor for the increasing, decreasing, and preschool-limited trajectory (Cote et al. 2009; Feng et al. 2008; Sterba et al. 2007). The advantage of the current study is that we included symptoms of psychopathology of both parents, assessed at the time when they were expecting a child. While adjusting for symptoms of psychopathology of the mother, paternal psychopathology independently predicted increasing, decreasing and preschool-limited trajectories of anxiety and depression symptoms as well. Whereas maternal psychopathology can influence both the fetus and the further development of the child, paternal psychopathology can act as a stressor in the direct environment of the child (O'Donnell et al. 2014; Stein et al. 2014).

The development of children’s anxiety and depression symptoms was associated with lower self-esteem and poorer school-related outcomes at the age of 10. In line with previous studies (Fanti and Henrich 2010; Sterba et al. 2007), children in the low group had more self-esteem, better school performance, and fewer school problems. More favorable outcomes were also found for children in the decreasing group, although the study by Feng et al. (2008) showed that boys with high declining symptoms of anxiety were more likely to be diagnosed with an anxiety disorder. As the decreasing trajectory was predicted by low maternal education and non-Dutch ethnicity of the child, a part of these children in this trajectory may grow up in a high-risk environment. Instead of persistent vulnerability, this group might be able to show resilience instead of persistent vulnerability when their environment becomes more enriched (Zolkoski and Bullock 2012). These results could also be explained by acculturation of these families over time (Sam and Berry 2010). Additional explorative analyses showed that for families with a non-Dutch ethnicity in the decreasing trajectory, a positive association was found between time in the Netherlands for mothers and self-esteem reported by children.

Both increasing and preschool-limited symptoms were associated with lower self-esteem and poorer school-related outcomes. Specifically, children who experienced consistent increasing symptoms reported the lowest levels of self-esteem with large effect sizes. As symptoms in the increasing trajectory reached borderline clinical levels at age 10, increasing anxiety and depression symptoms together with poorer functioning indicate that these children may be at risk of developing clinical problems. Both anxiety and depression during childhood and adolescence have been associated with diminished social skills and victimization (de Lijster et al. 2018; Kingery et al. 2010; Maughan et al. 2013). Internalizing and externalizing problems have previously been related to diminished social competence in middle childhood (Bornstein et al. 2010), which could explain why experienced friendship quality no longer differed between the trajectories after adjusting for external problems. It should also be mentioned that apart from the outcome self-esteem, effect sizes were low, which is fairly common in a sample that is representative of the general population.

### Strengths and Limitations

One strength of the current study is that we identified predictors of anxiety and depression symptom trajectories that were measured when parents were expecting a child. Also, this is the first study that relates anxiety and depression symptom trajectories to broader psychosocial and school functioning. Moreover, we related these trajectories to the children’s own reports of their experienced friendship and feelings of self-worth. However, the use of shorter versions of these questionnaire resulted in a lower internal consistency of these outcomes. Therefore, replication of associations between the trajectories and outcomes is warranted in studies that use more extensive measures of children’s psychosocial and school functioning. A final strength is that most previous studies failed to take the uncertainty of trajectory membership into account when the most likely assigned trajectory for each individual is exported to other statistical software outside the growth mixture model. By conducting all analyses within the growth mixture model, our results are not hampered by possible misclassifications of class membership.

One limitation of the current study, and in line with previous studies, is that we only relied on reports of the primary caregiver for measuring child anxiety and depression symptoms. In addition, average scores of the Anxious/Depressed subscale 1.5–5 years and 6–18 years consisted of a different number of items which may hamper the comparability of the average scores across time. As already mentioned, internal consistency of the Anxious/Depressed subscale was low at the age of 1.5 years. Comparable reliability coefficients less than 0.70 have been found for most narrow-band scales of the CBCL in preschool children world-wide (Rescorla et al. 2011). Moreover, the factor structure of the CBCL provides culture-general taxonomic constructs (Ivanova et al. 2010) and the Anxious/Depressed subscale has criterion-related validity in preschool children, despite low internal consistency (Achenbach and Rescorla 2000). In our study, the same trajectory solution appeared when performing the analyses without this first measurement. Future studies may consider using a Factor Mixture Model with item-response information to identify item-level latent variables of the CBCL. Another limitation is the number of children for whom no outcomes measures were available. We should be cautious when generalizing the trajectories as children with a non-Western ethnicity, mothers with a low level of education, and parents who reported on levels of psychopathology during pregnancy were underrepresented in the outcome samples because of drop-out. In particular, missing data on paternal psychopathology was high which weakens our confidence in the results of this predictor. As previous research has shown that study drop-out does not result into different associations between predictors and outcomes in population-based longitudinal follow-up studies (Wolke et al. 2009), we are hesitant to speculate whether these findings would have been different for the whole cohort.

### Implications for Prevention Policies

The current study has implications for prevention policies that aim to ward off problems with internalizing, psychosocial, and school functioning for young children in the general population. Our results showed that 7.4% of the children developed anxiety and depression symptoms within the borderline clinical range at age 10 and had lower self-esteem and poorer school-related outcomes. Our findings suggest that selective (i.e. asymptomatic population at higher risk) and indicated (i.e. high risk children with detectable symptoms foreshadowing clinical anxiety or depression) prevention programs are needed instead of universal prevention programs (Stockings et al. 2016; Vázquez-Bourgon et al. 2013; Werner-Seidler et al. 2017). In this study, parental psychopathology served as a risk factor for the development of children’s anxiety and depression symptoms. Although the risk to develop internalizing problems for children of parents with a mood or anxiety disorder is well known (Maciejewski et al. 2018; Weissman et al. 2016), general psychopathology should not be overlooked. Moreover, information about psychopathology experienced by the father should be taken into consideration as well. Prevention programs often target factors that are related to the maintenance of anxiety and depression symptoms instead of modification of risk factors (Lawrence et al. 2017). Selective and indicated prevention programs could benefit from addressing risk factors that predict the development of anxiety and depression symptoms, such as parental psychopathology or indicators of socioeconomic status.

## Conclusion

There are distinct courses of anxiety and depression symptoms in early childhood that are related to differences in self-esteem and school-related outcomes by the age of ten. These trajectories were predicted by child and family factors that are identifiable before anxiety and depression symptoms developed, and could, therefore, guide monitoring these symptoms in the general population and provide targets for prevention programs.
